# Endocrine Regulation of Extra-skeletal Organs by Bone-derived Secreted Protein and the effect of Mechanical Stimulation

**DOI:** 10.3389/fcell.2021.778015

**Published:** 2021-11-24

**Authors:** Yuxiang Du, Lingli Zhang, Zhikun Wang, Xuan Zhao, Jun Zou

**Affiliations:** ^1^ School of Kinesiology, Shanghai University of Sport, Shanghai, China; ^2^ School of Physical Education and Sports Science, South China Normal University, Guangzhou, China

**Keywords:** bone, secreted protein, cross-organ regulation, endocrine regulation, exercise prescription, mechanical stimulation

## Abstract

Bone serves as the support for body and provide attachment points for the muscles. The musculoskeletal system is the basis for the human body to complete exercise. Studies believe that bone is not only the basis for constructing structures, but also participates in the regulation of organs outside bone. The realization of this function is closely related to the protein secreted by bone. Whether bone can realize their positions in the human body is also related to their secretion. Bone-derived proteins provide a medium for the targeted regulation of bones on organs, making the role of bone in human body more profound and concrete. Mechanical stimulation effects the extra-skeletal organs by causing quantitative changes in bone-derived factors. When bone receives mechanical stimulation, the nichle of bone responds, and the secretion of various factors changes. However, whether the proteins secreted by bone can interfere with disease requires more research. In this review article, we will first introduce the important reasons and significance of the in-depth study on bone-derived secretory proteins, and summarize the locations, structures and functions of these proteins. These functions will not only focus on the bone metabolism process, but also be reflected in the cross-organ regulation. We specifically explain the role of typical bone-derived secretory factors such as osteocalcin (OCN), osteopontin (OPN), sclerostin (SOST) and fibroblast growth factor 23 (FGF23) in different organs and metabolic processes, then establishing the relationship between them and diseases. Finally, we will discuss whether exercise or mechanical stimulation can have a definite effect on bone-derived secretory factors. Understanding their important role in cross-organ regulation is of great significance for the treatment of diseases, especially for the elderly people with more than one basic disease.

## Introduction

The bones form the outline of the body, provide attachment points for muscle and serve as a support for the body physically. But bone is not generally considered an endocrine gland. The definition of endocrine gland is that it must be highly vascularized to form a system and directly secrete hormones into the blood so as to affect distant targets. According to this definition, the osteocyte lacunar contains bone fluid of bone cells release factors which can be found in circulation. It has the role of supporting and remodeling, also keep closely with the balance and maintenance of multiple trace elements in the human body. In adult bones, osteoblasts may account for approximately 5% of bone cells. Compared with 1% of osteoclasts, 90–95% are bone cells. Osteoblasts can release factors such as osteocalcin. In addition, bone cells can produce circulating factors such as FGF23 and SOST. With the gradual deepening of research, study showed that bone has the function of secreting protein factors, and believe that bone is an important endocrine organ (Brunetti et al., 2017; DeLuccia et al., 2019; Gomarasca et al., 2020). In recent years, it was found that bones are the largest secretory organs in the whole body, and secretory factors affect organs outside the bones through bone-derived factors including FGF23, prostaglandin E2 (PGE2), transforming growth factor-β (TGF-β), OCN and SOST. The structure and function of bone-derived secreted protein can be divided into two parts according to their positions. Intraosseous one can regulate the balance between bone formation and resorption. In addition to changes happened in its own bone microstructure and bone mass, the changes of its secretion factors will inevitably affect the external organs. Once they turn into extraosseous one, the function will be affecting extra-skeletal organs. These organs involve in the nervous system, glucose and lipid metabolism, blood cardiovascular system, muscles, thyroid and so on. The effect is of great difference.

The motor system includes bones, bone connections and skeletal muscles. Under the innervation of the nerve, the muscle contracts and pull the bone to which it is attached. Bone will respond when it receiving mechanical stimulation (or exercise) by changing its bone secretion. What we can determine till now is that mechanical stimulation (or exercise) will have an effect on bone-derived secreted proteins, and this impact will continuous exist for a short term. Mechanically sensitive cells can perceive mechanical stimuli through receptors on the envelope, such as primary ciliary complexes, integrins, and Ca^2+^ channels. For bone cells, the lacuno-canalicular network (LCN) can be used to quickly transmit signals. When the mechanical stimulus changes, the system responds quickly, generating fluid shear force on the surrounding cells. This change can affect the up-regulation of SOST and RANKL. The complex tensile and compressive stresses generated by mechanical stimulation can reduce the expression of SOST in bone cells and promote the process of bone formation and mineralization. For osteoclasts, the application of mechanical load stimulation can inhibit osteoclast differentiation, and the formation of osteoclasts is promoted after the stimulation is removed. Mechanical stimulation signals can induce osteoprotegerin (OPG) and inhibit RANKL to reduce osteoclast differentiation. Mechanical stimulation can change the bone niche, thereby changing the secretion of bone-derived proteins.

The protein factors secreted by bones not only participate in the metabolic process of the bone itself, but also communicate and regulate information with various organs to jointly achieve and maintain balances in the human body. From this point of view, bone-derived secretory factors should not only be protein factors involved in bone metabolism. These factors are important mediators for bones to regulate extra-skeletal organs. These proteins will specifically act on different target organs, depending on the distribution of their receptors. Not all bone-derived secreted proteins have the same properties.

This article analyzes the structures and source characteristics of common bone-derived secreted proteins to understand their basics of biology. Summarize their regulatory effects in extra-skeletal organs and their manifestations in diseases. For brain and neural network, OPN and OCN can have different effects on Alzheimer’s Disease (AD) patients. A variety of bone-derived secretory proteins can play a role in glucose and lipid metabolism, the target organs include liver, kidney, pancreas and so on. In the cardiovascular system, SOST, OPN and FGF23 can affect the state of blood vessels and change the impact of blood on the vessel wall. In the musculoskeletal system, these proteins can regulate inflammatory factors, cardiomyocytes and chondrocytes through specific pathways. Because bone-derived proteins can act on thyroid, they mainly interfere with thyroid bone diseases and hormone regulations. Mechanical stimulation has a regulatory effect on bone-derived proteins, but whether this effect can be used to treat diseases through specific intervention remains to be studied.

## The Location, Structure and Function of Bone-Derived Secreted Protein

Bone is now considered to be an endocrine organ. Bone cells have the ability to secrete and release protein factors. But there are still some differences among the factors, for example, the secretory cells. Some proteins are secreted by specific cells, while others can be derived from multiple cells. Therefore, the differences also exist in their structures and functions. Typical proteins secreted by specific cells include SOST secreted by osteocytes, FGF23 secreted by osteocytes and osteoblasts, OCN secreted by osteoblasts. At the same time, the proteins secreted and expressed by a variety of cells include OPN, PGE2 and TGF-β.

### Proteins Secreted by Specific Cells

#### Sclerostin

SOST is a glycoprotein secreted by mature bone cells. It is an inhibitor of Wnt signaling and bone morphogenetic protein (BMP), which can negatively regulate bone formation. For development and maintenance of bone, it plays an important role (Wang et al., 2018). The molecular weight of SOST is about 22kD. It is a secreted glycoprotein with cystine knot structure, including a signal sequence for secretion and two putative glycosylation sites. The cystine knot is a finger-like structure formed by two pairs of (Vázquez-Sánchez et al., 2021) twisted anti-parallel β chains. After SOST is secreted, it will anchor on the low-density lipoprotein receptor-related protein-4 (LRP4) receptor of the osteoblast membrane, so that SOST can be retained in the bone cavity. When SOST binds to the receptor LRP5/6 of osteoblasts, it will inhibit the downstream cascade of Wnt/β-catenin signaling in the cell through competitive binding (Xiong and O'Brien, 2012). SOST can do effect on inhibiting bone formation and negatively regulating Wnt/β-catenin signaling pathway.

#### Fibroblast Growth Factor 23

FGF23 is a hormone-like protein secreted by osteoblasts and osteocytes. It is a bone-derived factor that regulates the mineralization of extracellular matrix and a systemic hormone that participates in mineral metabolism. The length of FGF23 is more than 8.5 kb. The FGF23 gene is located on chromosome 12 for human and chromosome 6 for mouse, contains 3 exons (Goetz and Mohammadi, 2013). Hormone-like FGF23 has a poor affinity with heparan sulfate. It can be secreted from cells and diffuse into blood, circulating to target cells in distant organs (Asada et al., 2009). The co-receptor α-Klotho is on the surface of target cells for FGF23. The interaction between FGF23 and α-Klotho depends on the carboxyl end of FGF23 (Urakawa et al., 2006). The expression of FGF23 is regulated by many factors, such as 1,25 Dihydroxyvitamin D. It can promote the expression of FGF23 by activating VDR. At the same time, FGF23 can also inhibit the production of 1,25 dihydroxyvitamin D (Yu et al., 2005).

#### Osteocalcin

OCN is a non-collagenous acid glycoprotein which is synthesized and secreted by osteoblasts in bones (Gallop et al., 1980). It is a kind of calcium-binding protein that depends on vitamin K and is the main component of bone matrix. The relative molecular mass of osteocalcin is about 6kD. It has three γ-carboxyglutamate fragments at three positions of the peptide chain, 17, 21, and 24, which has a high affinity for calcium ions. The gene structure of OCN for both human and rat contains 4 exons and 3 introns (Hauschka et al., 1975). OCN is composed of an unstructured N-terminal, C-terminal hydrophobic core and 3 alpha helices (Zoch et al., 2016). Through γ-glutamyl carboxylase (GGCX) as catalysis, OCN will occur carboxylation reactions under acidic conditions. The difference in the completion of reactions will lead to different products. Undercarboxylated osteocalcin (ucOCN) is the active form of OCN. The protein structure of ucOCN after the removal of the propeptide contains 0–2 γ-carboxyglutamate residues. Carboxylated osteocalcin (cOCN) is inactive and mainly stored in bone to form the skeleton structure.

### Proteins Secreted by a Variety of Cells

#### Osteopontin

OPN is a non-collagen protein secreted by bone cells, osteoblasts, osteoclasts and other cells. It belongs to small integrin-binding lIgand, n-linked glycoproteins (SIBLING) and is an important component for regulating the mineralization of extracellular matrix (De Fusco et al., 2017). The length of OPN is about 8 kb. The OPN coding gene is located on chromosome 4 for human and chromosome 5 for mouse (Bouleftour et al., 2019). It contains 7 exons and 6 introns. The C-terminal of OPN binds two heparin molecules and the CD44 variant, the N-terminal contains the binding region of the integrin receptor (Han et al., 2019). OPN is related to osteoblast mRNA of bone morphogenetic protein signaling pathways (BMPS) downstream, which can stimulate the proliferation and calcification of osteoblasts. It is also a pro-inflammatory cytokine that can regulate migration and communication of immune cells, also response to brain injury. It plays an important role in many kinds of neuroinflammatory diseases (Sun et al., 2013).

#### Prostaglandin E2

PGE2 is one of the diverse prostaglandins (Narumiya et al., 1999), which composed of 378 amino acids. The relative molecular mass of PGE2 is about 43. It is one of the metabolites of arachidonic acid (Wang and Dubois, 2010). The synthesis and catabolism of PGE2 require the participation of multiple enzymes. PGE2 is a hormone-like chemical messenger, which is rapidly oxidized in the body. With an extremely short half-life, it only plays a role in the vicinity of synthetic cells after being released (Tai et al., 2002). By binding to its receptors, it activates and transduces the corresponding signaling pathways in the cells to achieve biological functions. After PGE2 binds to the E-type prostaglandin receptor, which is a G protein-coupled receptor. It will stimulate a variety of downstream signaling pathways (Hatae et al., 2002). Among which the most classic signaling pathway is Wnt/β-catenin. PGE2 can promote regeneration by improving the stability of β-catenin to increase the activity of the pathway (Zhu et al., 2017). This is different from the negative regulation effect of SOST.

#### Transforming Growth Factor-β

TGF-β is a polypeptide signaling molecule, the superfamily of which includes more than 40 structurally related factors. TGF-β has a wide range of regulatory effects on cell functions, including regulating growth and maintaining the balance of the internal environment (Ismaeel et al., 2019). It can stimulate fibroblasts to synthesize collagen and fibronectin, promoting their deposition in the extracellular matrix. It has a strong chemotactic effect on inflammatory cells and fibroblasts, which can enhance local inflammation. In addition, it also has the function of enhancing the synthesis of denervated skeletal muscle myogenic stem cells and increasing the secretion of extracellular matrix. This process will promote the fibrosis of denervated skeletal muscle. According to research investigations, there are currently known that TGF-β has at least 6 isomers. Mammalian TGF-β mainly includes TGF-β1, TGF-β2, and TGF-β3 subtypes. The three genes of human are respectively located on chromosomes 19q3, 1q41 and 14q24 (Baugé et al., 2014). The nucleotide sequence of each subtype is highly homologous. All exist in the form of homodimers, containing 7 exons. These three TGF-β subtypes are expressed in chondrocytes.

## Cross-Organ Regulation of Bone-Derived Secreted Protein

### Brain and Neural Network

#### Osteocalcin Affects Cognitive Ability by Binding to Receptors in the Brain

The function of OCN is diversified. It can be divided into two parts: internal and external bones. OCN can indicate the efficiency of bone turnover and take part in the formation of skeletal structure (Vlot et al., 2018). Carboxylated osteocalcin (cOCN) can locate and adsorb hydroxyapatite, which is the basis for maintaining normal bone mineralization. OCN is only synthesized and secreted by mature osteoblasts and osteocytes, then expressed under the control of Runx2/Cfa1 transcription factor. Therefore, its content in serum can indicate bone turnover efficiency and osteoblast activity. As one of the biochemical indicators that specifically reflect bone formation, it can be used for the diagnosis of bone-related diseases. It can also used to check bone condition and observe the effect of intervention treatment. It also has the characteristics of hormones. The active ucOCN can regulate extraosseous organs.

**FIGURE 1:**
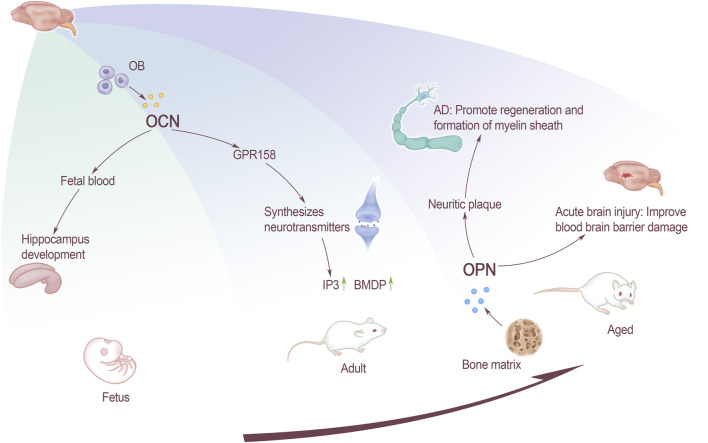
The effect of bone-derived factors on brain at all stages. OCN affects the development of fetal hippocampus through fetal blood, and the GPR158 receptors that acted on adults affects the synthesis of neurotransmitters. For elderly, OPN affects inflammatory plaques and promotes the remyelination and formation of AD patients. Improve the function of the blood-brain barrier in patients with acute brain injury.

Patients with AD are more likely to get osteoporosis than normal people (Rousseaud et al., 2016). OCN is present in the blood of the fetus during the pre-embryonic period when the fetus undergoes skeletal development. The OCN content of pregnant mice has an effect on the normal development of the fetus’ brain, and the benign effect can help the hippocampus development. Knockout of the mother mouse OCN will lead to apoptosis of some fetal nerve cells, and even affect the learning and memory-like behaviors of adult offspring. OCN can cross the blood-brain barrier and bind to G protein-coupled receptor 158 (GPR158) on neurons in the brainstem, midbrain, and hippocampus. GPR158 has been clearly confirmed to be an OCN receptor in brain (Kosmidis et al., 2018), which binds in the CA3 area (Lee et al., 2007), promotes inositol triphosphate (IP3) and brain-derived neurotrophic factor (BDNF) accumulation. G protein-coupled receptor Class C Group 6 Member A (GPRC6A) does not express OCN signals in brain, but OCN can affect cognitive performance through the expression of other receptors (Obri et al., 2018). Therefore, OCN uses a variety of receptors to achieve its functions and play an important role of regulatory in the central nervous system. Although OCN works in conjunction with neurons in the dorsal brainstem and the median sulcus, GPR158 is not expressed in this area. Therefore, it is speculated that OCN may also have a third receptor. This speculation supports the previous view that multiple receptors work (Figure 1).

In the process of aging, improving bone health can do good effect on cognitive function. Exercise can have a positive impact on mind, such as preventing AD and so on. Whether the change of OCN can correct AD caused by aging needs further research to know. As a new part of endocrine organs, more and more researches have focused on the influence of OCN on brain, including the regulation pathways of the nervous system, the effect on memory formation and so on.

The influence of OCN on the nervous system can throughout body’s life. From the fetal period through the maternal blood affecting fetal brain development, to affect the neurotransmitter synthesis in adulthood, and then participating in the process of cognitive dysfunction and even cause AD. As a research hotspot of endocrine organs, the researches of bones are focusing on the effects of OCN on the brain. These include the regulation of brain development, pathways of nervous system, memory formation, and intervention in neurological diseases. However, OCN, especially ucOCN, as an important intervention method for the diagnosis and treatment of neurological diseases needs to be further improved.

#### Osteopontin Promotes Macrophage Migration and Pro-inflammatory Cytokine Production

OPN can inhibit the pathology of various brain diseases through neuroprotection and promotion of repair (Rentsendorj et al., 2018). Not only plays an important role in the extracellular matrix of the central nervous system (CNS), but is also a key factor for matrix remodeling and cell repairing after CNS injury. In addition, OPN plays a key role in traumatic brain injury, stroke, ischemia and neurodegenerative diseases, such as AD and other diseases. The expression of OPN in the brain is low under normal circumstances. It is mainly expressed in the olfactory bulb, cerebellum and brainstem neurons, and the expression in the pons and medulla is higher than that in the midbrain. But it is significantly up-regulated in the case of injury or inflammation (Zhou et al., 2020), such as acute brain injury. Activating the P42/44 MAPK and PI3/Akt pathway can lead to the synthesis of a new protein in the cell, and the PI3/Akt pathway plays a key role in cell apoptosis, blood-brain barrier destruction, neurogenesis and angiogenesis. After acute brain injury, endogenous OPN can improve the damage of the blood-brain barrier through different mechanisms.

Sun found through the rat model of intravascular perforation that OPN is associated with brain injury. The increase of endogenous OPN and autophagy-related proteins suggests that OPN may regulate the autophagy-apoptosis interaction by activating the function of autophagy to reduce early brain damage and inhibit neuronal apoptosis (Condomitti et al., 2018). In the case of cerebral hemorrhage, the expression of OPN will rise. This causes microglia and macrophages to be activated accordingly. By inducing the migration and proliferation of neuroblasts and promoting nerve regeneration, it accelerates the recovery of brain function (Yan et al., 2009). In the case of cerebral hemorrhage, the expression of OPN will increase and it will cause the activation of microglia and macrophages to induce neuroblasts to migrate and proliferate, which will accelerate the recovery of brain function (Yan et al., 2009).

AD is the most common neurodegenerative disease today. Its mainly pathological features are the accumulation of β-amyloid peptide (Aβ) and neurofibrillary tangles, which ultimately manifests as severe cognitive dysfunction. Studies have found that inflammation is closely related to Aβ precipitation. OPN is a molecule involved in the recruitment and activation of macrophages, and may contribute to the repair promotion process in the brain. Previous studies have suggested that without OPN, the migration of macrophages and the production of pro-inflammatory cytokines will be impaired. OPN can promote the formation and regeneration of myelin (Carecchio and Comi, 2011), it may play a role in the remodeling process related to abnormal neuron re-entry into the cell cycle in the brain of AD patients. OPN in brain tissues of AD rats are mostly concentrated on inflammatory plaques, and their expression increases. The staining intensity of OPN in the hippocampus is positively correlated with age and Aβ precipitation. It is suggested that the increase in OPN expression may be a manifestation of accelerated neurodegeneration and pathological changes (Wung et al., 2007; Liao et al., 2019). The occurrence of AD will cause a large number of neurons in the brain tissue to loss. Through cell culture *in vitro*, OPN was found to promote the formation and regeneration of myelin sheath (Le Guenno et al., 1990). It is speculated that OPN may play a role in the remodeling process associated with abnormal neuronal re-entry into the cell cycle in the brain of AD patients. However, current research shows that OPN’s effect on neurodegenerative diseases seems to be a double-edged sword, and there may be two different effects. On the one hand, OPN can act as a neuroprotective agent by up-regulating the formation of myelin sheath and remyelin sheath. On the other hand, OPN may play a role in accelerating the disease by triggering neuronal toxicity and apoptosis (Sun et al., 2013) (Figure 1).

### Glucose and Lipid Metabolism

#### Osteocalcin Maintains the Body’s Glucose Homeostasis and Reduces Fat Accumulation

The lackness of OCN may lead to decreases in the number of islet β cells, insulin content and an increase in blood glucose. As an OCN receptor, GPRC6A can mediate its corresponding functions in the endocrine system, including regulating and maintaining glucose homeostasis. For example, the loss of GPRC6A receptor will cause glucose intolerance (Lee et al., 2007). Increasing OCN can promote the proliferation of islet β-cells and insulin secretion, also enhance insulin sensitivity. Decreasing or even lack of OCN will cause pancreatic islets to shrink, decrease t islet β-cells and insulin.

Regarding the interaction between osteoblasts and insulin, studies have proposed the mechanism of “skeleton-pancreas feedback loop”. When insulin content rises, it can directly stimulate osteoblasts and promote their differentiation, increasing OCN content. If the insulin content decreases, it will reduce the number of osteoblasts, resulting in decreased OCN activity, affecting the process of bone formation and bone turnover. Insulin acting on the corresponding receptors in osteoblasts can also inhibit OPG expression and increase osteoclast activity. Create an acidic environment in the matrix for the conversion of OCN to active ucOCN. The liver is the central organ of energy metabolism, and the concentration of commonly used indicators of liver damage is negatively correlated with the level of OCN. OCN can be used as an independent indicator to judge the degree of ballooning of non-alcoholic steatohepatitis.

OCN can also reduce non-alcoholic steatohepatitis by decreasing the expression of pro-inflammatory factors and pro-fibrotic genes. It can improve liver cell steatosis, degeneration and fibrosis caused by ballooning (Chamouni et al., 2015). Due to abnormal bile secretion, patients with primary biliary cirrhosis will reduce OCN secreted by osteoblasts (Gupte et al., 2014). OCN can play a key role in the formation and activation of GPRC6A receptors in adipocytes. When ucOCN binds to the GPRC6A receptor in adipose tissue, it participates in energy metabolism. It changes liver fat and triglyceride levels, too. By increasing energy consumption and promoting metabolism, ultimately make weight loss achieved (Condomitti et al., 2018). OCN secreted by bones acts on adipose tissue, promotes the secretion of Leptin (LEP) and adiponectin (APN) from adipose tissue.

OCN regulates glucose and lipid metabolism through LEP, so that bones are believed to affect fat metabolism by controlling appetite. The interaction between OCN and APN is closely related to insulin sensitivity, obesity and serum triglyceride levels. First of all, OCN can send a feeding saturation signal to the hypothalamus of the feeding center through the interaction of LEP and serotonin to suppress appetite, reducing fat intake and affecting glucose and lipid metabolism. Secondly, OCN reduces body fat content by inducing the expression of APN in adipocytes. Esp−/− mice will not develop into obesity or diabetes. Overexpression of APN can enhance insulin sensitivity. Therefore, increasing the expression of APN can improve insulin sensitivity, promote fat metabolism, reduce the levels of free fatty acids and triglycerides in the body, and ultimately reduce body weight. Changes in the content of adipokines will in turn affect the OCN content. On the one hand, LEP binds to receptors in the hypothalamus and brainstem through the blood-brain barrier, and sends a signal to prevent the synthesis of serotonin. By inhibiting the release of serotonin from the midnuclear suture, it reduces sympathetic nerve activity. This leads to increased bone resorption and inhibition of bone formation, which in turn affects the content of active OCN. On the other hand, APN can bind to Adiponectin receptor protein 1 (AdipoR1) and activate intracellular signaling pathways. The increase of APN content enhences the expression of APN on the surface of osteoclast precursor cells, which affects the differentiation and maturation of osteoclast, in turn affects OCN content.

#### Sclerostin Provides Reference for the Diagnosis of Liver and Kidney Disease

Wnt signaling may have a potential role in chronic kidney disease (CKD). Chronic kidney disease-mineral bone disorder (CKD-MBD) is an abnormal mineral metabolism caused by chronic kidney disease. This disease is characterized by renal dystrophy, calcification of blood vessels and soft tissues. As the glomerular filtration rate (GFR) decreases, the concentration of SOST increases. In patients with end-stage renal disease (ESRD), the circulating level of SOST can reach 2–4 times that of the normal population. Kidney transplantation is the choice for patients with CKD in the end-stage disease. Studies have found that elevated serum SOST levels are an independent risk factor for death in kidney transplant patients (Zeng et al., 2018). The serum SOST level of CKD patients is higher than that of the general population, and it gradually increases as the level of renal function deteriorates. Decreased renal function and the occurrence of osteoporosis in the elderly may be related to the increase in SOST. SOST was detected in the proximal tubular cells, indicating that SOST is actively reabsorbed from urine. Increased renal excretion of SOST in CKD patients may be due to increased SOST production and decreased tubular reabsorption. However, whether the inhibition of SOST can prevent the bone loss or vascular calcification of CKD remains to be further studied. The detection of serum SOST can be used as a biomarker of bone metabolism disorders in patients with end-stage renal disease (Maré et al., 2019). There is a negative correlation between serum SOST and PTH in patients with end-stage renal disease, and PTH can be used as a regulation of serum SOST. SOST level can provide a reference for the severity of nephritis and other diseases, elevated serum SOST is a risk factor for kidney disease (Wakolbinger et al., 2020).

Osteoporosis is a common complication in patients with chronic hepatitis. About 75% of patients with chronic liver disease will suffer from osteoporosis. The disease affects the patient’s quality of life and increases the patient’s risk of fracture. Cirrhosis is the end-stage manifestation of chronic liver disease, and the SOST level of patients is higher than that of healthy people. SOST is negatively correlated with serum albumin, which is a marker of liver dysfunction. Moderate or severe liver dysfunction will affect the level of serum SOST. Alcoholism is an important inducing factor of osteoporosis, 35.9% of patients with alcoholic liver disease have changes in bone metabolism and structure. Patients with alcoholic liver disease have lower SOST levels, which may be caused by alcohol promoting bone cell apoptosis. The main site of increased SOST in patients with primary biliary cirrhosis (PBC) is the bile duct (Ehnert et al., 2019), and the level of SOST in serum is related to the decrease of BMD. Regulating SOST levels through blood circulation will affect bone metabolism, which will help improve osteoporosis in patients with liver cirrhosis. The serum SOST level of patients with early PBC showed a downward trend with time. In the later stage of liver disease, bile acids gradually accumulate, and bilirubin reduces the mitochondrial activity of bone cells. SOST in the serum of the bile nodules will affect the proliferation ability of bone cells. The bile and serum of patients with macula will reduce the survival and mineralization ability of bone cells, and the increase of SOST will affect bone cells in the process of bile formation (Guañabens et al., 2016).

#### Prostaglandin E2 Promotes DNA Synthesis in Liver Cells and Affects Fat Production

PGE2 is involved in the regulation of fat metabolism (Yan et al., 2009) and has the function of promoting regeneration of liver tissues (Stock et al., 2001). Prostaglandin E synthases (PGESs) and corresponding receptors are highly expressed in white fat, they are closely related to the occurrence of liver diseases. PGESs includes the following three types, microsomal prostaglandin E synthase (mPGES)-1, mPGES-2 and cytosolicprostaglandin E synthase (cPGES) (Miyoshi et al., 2017). These PGESs directly participate in the synthesis of PGE2 and affect liver diseases such as NAFLD, NASH, DILI and liver cancer. PGE2 has influence on fat with the participation of the receptor EP4. EP4 is a G protein-coupled receptor with 7-pass transmembrane structure, which mainly distributed in white fat (Hara et al., 2010). Activation of the EP4 receptor will inhibit the differentiation of adipocytes (Madden and Morrison, 2004), and lipid breakdown can be promoted by the lack of this receptor (Inazumi et al., 2011).

PGE2 has the effect of promoting DNA synthesis in liver cells, the effect is related to the concentration of PGE2. The increase of PGE2 is synchronized with the increase of cAMP in hepatocytes. PGE2 can change the stability of β-catenin through the phosphorylation process mediated by cAMP/PKA signaling pathway. By regulating the activity of the Wnt signaling pathway, liver regeneration is promoted. Inhibition of PGE2 synthesis will block Wnt-induced liver regeneration. The binding pathway of PGE2 and EP4 receptor can protect liver function, the use of EP4 selective agonist will reduce the degree of liver ischemic damage.

#### Osteopontin Affects Kidney Stones and Changes the Cycle of Cancer Cells

In the kidneys of healthy adults, OPN is mainly expressed in the thick ascending branches of the ring of Henle. In recent years, OPN has been reported in the study of kidney stones, kidney cancer and other related kidney diseases (Xie et al., 2001). The expression is regulated by many factors, such as parathyroid hormone, calcium, phosphorus, tumor necrosis factor α (TNF-α), transforming growth factor β1 (TGF-β1), epithelial growth factor and so on.

In kidney stone disease, OPN not only plays a key role in regulating the nucleation, growth and aggregation of calcium oxalate (CaOx), but also affects the adhesion of CaOx to renal epithelial cells, participating in the reservation of kidney stone. The main component of kidney stones is CaOx crystals. OPN, as the main crystal regulator, is considered to be one of the most important macromolecules affecting mineralization and kidney stone formation (Tsuji et al., 2014). However, whether OPN plays a role in inhibiting or promoting the formation of stones is still a controversial issue. As the urine form of OPN, urinary calcin can induce the formation of calcium oxalate dihydrate (COD) by inhibiting the accumulation of calcium oxalate monohydrate (COM). This can reduce the growth and aggregation of CaOx, prevent the combination of crystals and renal epithelial cells, and ultimately protect the kidneys from the deposition of calcium oxalate crystals.

Kidney cancer is one of the most common cancers in the world. OPN plays a key role in the growth and invasion of renal cancer. On the one hand, it may inhibit the apoptosis of cancer cells, promote the growth of tumor cells. On the other hand, it may provide favorable conditions for tumor cell tissues to occurrence and metastasis by inducing urokinase-type plasminogen activator (UPA) (Ghorbani et al., 2015).

#### Fibroblast Growth Factor 23 Is Related to G-3-P and can Promote Renal Phosphate Excretion

As a bone-derived hormone-like protein, FGF23 also plays an important role in regulating metabolism of multiple organs. Among them, the most widely studied is the regulation of FGF23 on the kidneys. FGF23 reaches kidney after it is secreted, and stimulates the excretion of phosphate in the urine by inducing the endocytosis of sodium-phosphate cotransporters NPT2a and NPT2c on the root tip of the proximal tubule cells of the kidney (Kaleta, 2019).

Elevated FGF23 levels are an early progressive and common complication of chronic kidney disease (CKD). Elevated FGF23 in CKD patients can promote renal phosphate excretion and help delay the onset of hyperphosphatemia. But it can cause a large number of compensatory harms, including calcitriol deficiency, changes in calcium homeostasis, and secondary hyperparathyroidism (Gutierrez et al., 2005; Musgrove and Wolf, 2020). In addition, the high level of FGF23 in CKD patients can cause pathological left ventricular remodeling, atrial fibrillation and heart failure, the risk of infection and death will increase. In mice and CKD patients, FGF23 elevated inhibits the activation, adhesion and transepithelial migration of neutrophils. Thereby reducing the recruitment of neutrophils and host defense during inflammation (Rossaint et al., 2016). In acute kidney injury (AKI), the level of FGF23 will also increase immediately, which shows that certain factors are produced in the kidney to promote the production of FGF23.

Existing studies use proteomics and metabolomics to analysis proteins and metabolites related to arterial FGF23 levels which screened out from renal venous blood of patients undergoing cardiac catheterization. It was identified that renal vein glycerol-3-phosphate (G-3-P) has a significant correlation with FGF23 (Simic et al., 2020). Renal vein G-3-P has a significant correlation with FGF23 (Simic et al., 2020). G-3-P can affect related physiological phenotypes caused by changes of FGF23. Circulating G-3-P is locally converted to lysophosphatidic acid (LPA) in bone and bone marrow through G-3-P acyltransferase isoform 2 (GPAT2). LPA binds to LPA receptor 1 (LPAR1) on cells which secret FGF23 to stimulate the production of FGF23 (Simic et al., 2020). G-3-P is an intermediate metabolite in the process of glycolysis, lipogenesis and oxidative phosphorylation (Possik et al., 2017). But its function is not limited to this. It can regulate insulin secretion, the synthesis and storage of fat, and FGF23 production.

In CKD patients, iron deficiency and increased blood erythropoietin (EPO) levels can stimulate the expression of FGF23. In CKD patients and kidney transplant recipients, iron deficiency is an important determinant of total FGF23 levels, which has a significant impact on the progression and mortality of CKD (Eisenga et al., 2017; Eisenga et al., 2019).

### Blood and Cardiovascular System

#### Sclerostin Affects Atherosclerosis by Inhibiting Angiotensin II

Atherosclerosis is a clinical manifestation of vascular aging, mainly due to abnormal proliferation of vascular smooth muscle (VSMC). SOST exists in the atherosclerotic tissue, the main function of it is to inhibit the Wnt pathway by binding to the transmembrane Wnt core receptors LRP-4,-5, and-6. SOST is expressed in the thoracic and abdominal aorta of an arterial-calcification mouse model, which is induced by the inhibition of angiotensin II (Ang II). This expression plays an important role in the pathogenesis of aneurysms and atherosclerosis (Krishna et al., 2017).

The up-regulation of SOST can down-regulate the expression of OPN and OPG in the mouse aorta. OPN can promote inflammation and participate in the activation of arterial calcification in Ang II mice, increasing the activity of Matrix metalloproteinase-9 (MMP-9). The concentration of OPG is positively correlated with hemangioma, and it can promote the inflammatory response of vascular smooth muscle cells through cathelicidin S, Matrix metalloproteinase-2 (MMP-2) and MMP-9. SOST can inhibit the formation of aneurysms and atherosclerosis induced by Ang II, and regulating SOST can be used as a potential way to inhibit these diseases.

In patients with type 2 diabetes, the circulating SOST of those with atherosclerosis will increase. In male patients with atherosclerosis, the levels of serum sclerostin are positively correlated with aging, but this difference has not found in women (Morales-Santana et al., 2013). The high level of SOST is related to death caused by cardiovascular disease. Hyperglycemia, insulin resistance and other cardiovascular risk factors can cause vascular endothelial damage, which promotes vascular calcification. The increase of serum SOST in hemodialysis patients (Brandenburg et al., 2013) may be due to the increase of SOST produced by bone, it may also be caused by the decrease in renal clearance or the physiological adaptation of blood vessels as a result of increased calcification.

#### Osteopontin Is Involved in Vascular Calcification and Endothelial Hyperplasia

OPN mediates the process of cell adhesion, proliferation and migration, which is related to the pathophysiology of tumors. As one of the extracellular matrix (ECM), overexpression of OPN will promote cardiac fibrosis and participate in the remodeling process of the heart and blood vessels (Sawaki et al., 2018). In addition, it will also affect the formation of vascular calcification. The serum OPN level of patients with vascular calcification will be significantly increased, which is one of the signs of vascular calcification (Wolak, 2014). OPN affects the formation of atherosclerotic plaques in arteries, and elevated blood glucose levels will promote the expression of OPN in endothelial cells. Vascular remodeling refers to the thickening of the vessel wall and the increase of resistance in the vessel. OPN is associated with vascular remodeling and ventricular hypertrophy in hypertensive patients. After lowering blood pressure, the OPN level of patients decreases (Hou et al., 2014). Lack of OPN will inhibit the process of tissue remodeling, especially in the fibrosis process after myocardial infarction (Okamoto, 2007). In view of this fibrotic process, OPN is considered to be possible for treatment because it has the effect of promoting angiogenesis, wound healing and tissue regeneration (Abdelaziz Mohamed et al., 2019).

#### Fibroblast Growth Factor 23 Affects Nephropathy Secondary Cardiovascular Disease

FGF23 plays an important role in the regulation of vitamin D and blood phosphorus levels. Klotho, the receptor cofactor of FGF23, is very important in this metabolic regulation (Hu et al., 2010). Klotho deficiency is a biomarker of kidney disease, and it is also an important pathogenic factor for cardiovascular disease in chronic kidney disease such as myocardial fibrosis and vascular calcification (Sopjani et al., 2015). Increasing klotho levels can tireduce serum creatinine and urea nitrogen levels by indirectly regulating the homeostasis of phosphorus and calcium in the body. At the early stage of patients with chronic kidney disease, the decrease of Klotho level will cause a compensatory increase in FGF23. By inhibiting the reabsorption of phosphorus in the proximal renal tubules, it promotes urinary phosphorus excretion to maintain the balance of blood phosphorus metabolism. The compensatory increase of FGF23 can inhibit the level of 1,25-(OH)_2_D_3_ and induce parathyroid glands to continuously secrete PTH. This change will stimulate the bones to release phosphorus into the blood, causing blood phosphorus to increase, and aggravating disorders of calcium and phosphorus metabolism. If phosphate continues to stimulate bone tissue to secrete FGF23, kidney klotho secretion will decrease, and the compensatoryly increased FGF23 will also lose its effective regulation of bone minerals. Lead to the imbalance of bone mineral metabolism and accelerate the process of vascular calcification in chronic renal failure (Lu and Hu, 2017).

### Skeletal Muscle Movement System

#### Osteopontin Plays a Regulatory Role in Skeletal Muscle

**FIGURE 2:**
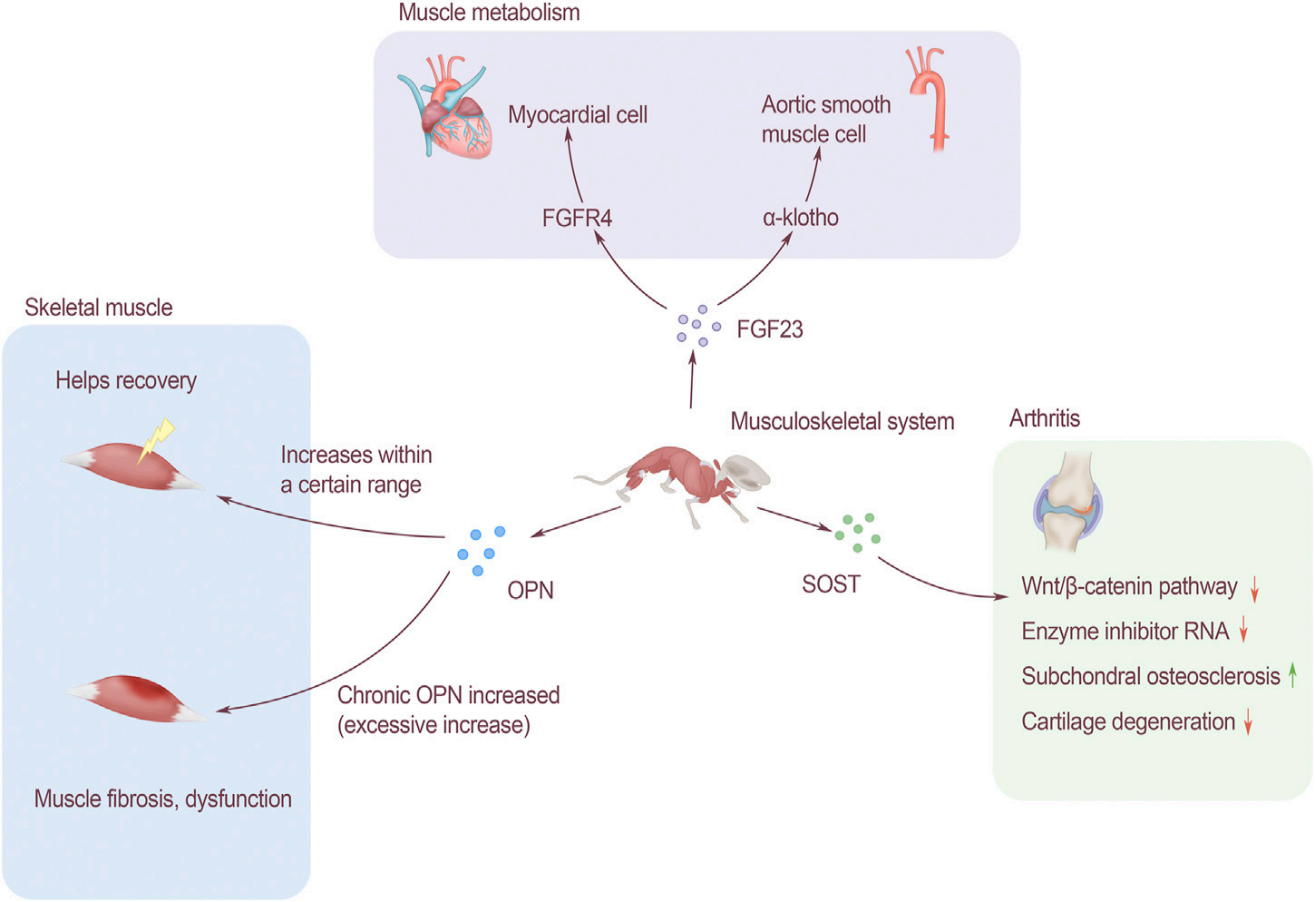
The effect of bone-derived factors on modules of musculoskeletal system. FGF23 affects muscle metabolism. It is associated with cardiomyocytes through FGFR4 and acts on α-Klotho to change aortic smooth muscle. OPN affects skeletal muscles, and the increase in a certain range after injury will help recovery, but long-term increase of OPN will lead to muscle fibrosis and affect muscle function. SOST affects arthritis through the Wnt/β-catenin signaling pathway, which can inhibit the activity of enzyme inhibitor RNA, promote subchondral bone sclerosis, and inhibit cartilage degradation.

OPN plays an important role in regulating the proliferation, differentiation and regeneration of skeletal muscle cells. Studies have shown that OPN is a key inflammatory cytokine of tissue remodeling^[63]^. Many types of inflammatory cells express OPN, including T cells, neutrophils and macrophages. The expression of OPN in normal muscles is relatively low, but when the muscles are injured, it will be approximately 120 times that of the baseline level within 12–24 h (Hoffman et al., 2013). OPN may support the rapid return of muscle function to normal in the early stage of injury (Uaesoontrachoon et al., 2013). Limited acute OPN overexpression is beneficial to damaged muscles, but chronic overexpression of OPN may lead to fibrosis and functional impairment of damaged muscles (Pagel et al., 2014). The expression of OPN is significantly increased in Duchenne muscular dystrophy (DMD) patients and muscular dystrophy (MDX) mice, suggesting that OPN is involved in muscle regeneration and ammoniation. It is currently known that OPN plays an important role in DMD and skeletal muscle injury, but its related functions and regulatory effects are still unclear (Figure 2).

#### The Role of Fibroblast Growth Factor 23 in Muscle Metabolism

Left ventricular hypertrophy (LVH) is a common pattern of cardiovascular damage among CKD patients, and 75% of them were founded LVH when they reached the end-stage of renal disease (Faul et al., 2011). The complex pathogenesis of LVH involves ventricular pressure and volume overload, and the bone-derived FGF23 plays an important role in it (Gutiérrez et al., 2009). FGF23 induces the hypertrophic growth of cardiomyocytes and the left ventricle of rodents through a mechanism directly dependent on FGFR, but does not depend on α-klotho receptors. In terms of molecular mechanism, FGF23 specifically activates FGFR4 on cardiomyocytes to activate phospholipase Cγ/calcineurin/nuclear factor of activated T signaling (PLCγ/calcineurin/NFAT signaling). Soluble Klotho is a circulating form of FGF23 receptor, which can prevent the effect of FGF23 on cardiomyocytes by increasing the expression of PDE3A and PDE3B (Lindner et al., 2020). Klotho is expressed in human arterial and aortic smooth muscle cells. In CKD patients, chronic circulating stress factors including uremic serum, high calcium, TNF-α and other components can inhibit the expression of Klotho in vascular smooth muscle. Therefore, when α-Klotho is missing, it will cause smooth muscle cell calcification and loss of response to FGF23 through Runx2 and muscle cell-serum response factor pathways (Huang et al., 2020). FGF23 and Klotho play an important role in the pathogenesis of vascular calcification in CKD patients (Figure 2).

#### Sclerostin Regulates Arthritis Process

SOST gene can be expressed by chondrocytes, and changing the activity of SOST will have an impact on articular cartilage. In osteoarthritis, the SOST of chondrocytes increases locally, but decreases in the subchondral bone. SOST can inhibit Wnt/β-catenin signaling, and can also reduce the RNA levels of key matrix components and enzyme inhibitors. SOST can regulate the process of osteoarthritis of bone and cartilage. Promoting the sclerosis of subchondral bone, at the same time inhibiting cartilage degradation.

As the age increases, the number of cartilage cells in animals decreases, and the same as the expression of SOST (Thompson et al., 2016). There was no significant change in the expression of SOST in the bone tissue of patients with knee osteoarthritis. Normally aging articular cartilage will not be affected by changes in SOST levels (Roudier et al., 2013). It is speculated that SOST may have nothing to do with cartilage destruction in knee osteoarthritis, or there are other compensation molecules in the cartilage, so the effect of SOST inhibition is masked (Figure 2).

### Thyroid and Related Hormones

#### Osteocalcin Affect Thyroid Bone Disease

The occurrence of thyroid bone disease is related to abnormal thyroid level (Cheng et al., 2021). Thyroid hormone has a non-genomic effect in osteoblasts of primary mouse (Asai et al., 2009). This effect can inhibit tyrosine kinase through thyroid hormone to stimulate OCN expression. Excessive or insufficient thyroid hormone can cause bone mineral loss, leading to the occurrence of osteoporosis. When the thyroid function of patients with hyperthyroidism returns to normal, the OCN level will significantly lower than that in the hyperthyroid stage (Ma et al., 2011).

Patients with abnormal levels of thyroid hormone have a chance of getting thyroid bone diseases. Among these diseases, osteoporosis is the most common. In the study of the relationship between OCN and thyroid, the treatment of osteoporosis may become the focus of research.

#### Regulation Between Fibroblast Growth Factor 23 and Parathyroid Hormone

PTH stimulates FGF23 serum levels indirectly by increasing the synthesis of 1,25-dihydroxyvitamin D. Loss of parathyroid glands in Gcm2 knockout mice led to a decrease in 1,25-dihydroxyvitamin D levels and FGF23 concentrations, and FGF23 levels will return to normal after 1,25-dihydroxyvitamin D injection (Liu et al., 2006). The parathyroid gland is one of the target organs of FGF23, mice that overexpressing FGF23 will get hyperparathyroidism (Bai et al., 2004). Recombination of FGF23 will increase the level of Klotho in the parathyroid glands. FGF23 activates the MAPK pathway of the parathyroid glands through the phosphorylation of ERK1/2 and increases the level of early growth response 1 (Erg1) mRNA. FGF23 can also inhibit secretion and gene expression of PTH(Ben-Dov et al., 2007).

## The Effect of Mechanical Stimulation on Bone-Derived Secreted Protein

**FIGURE 3:**
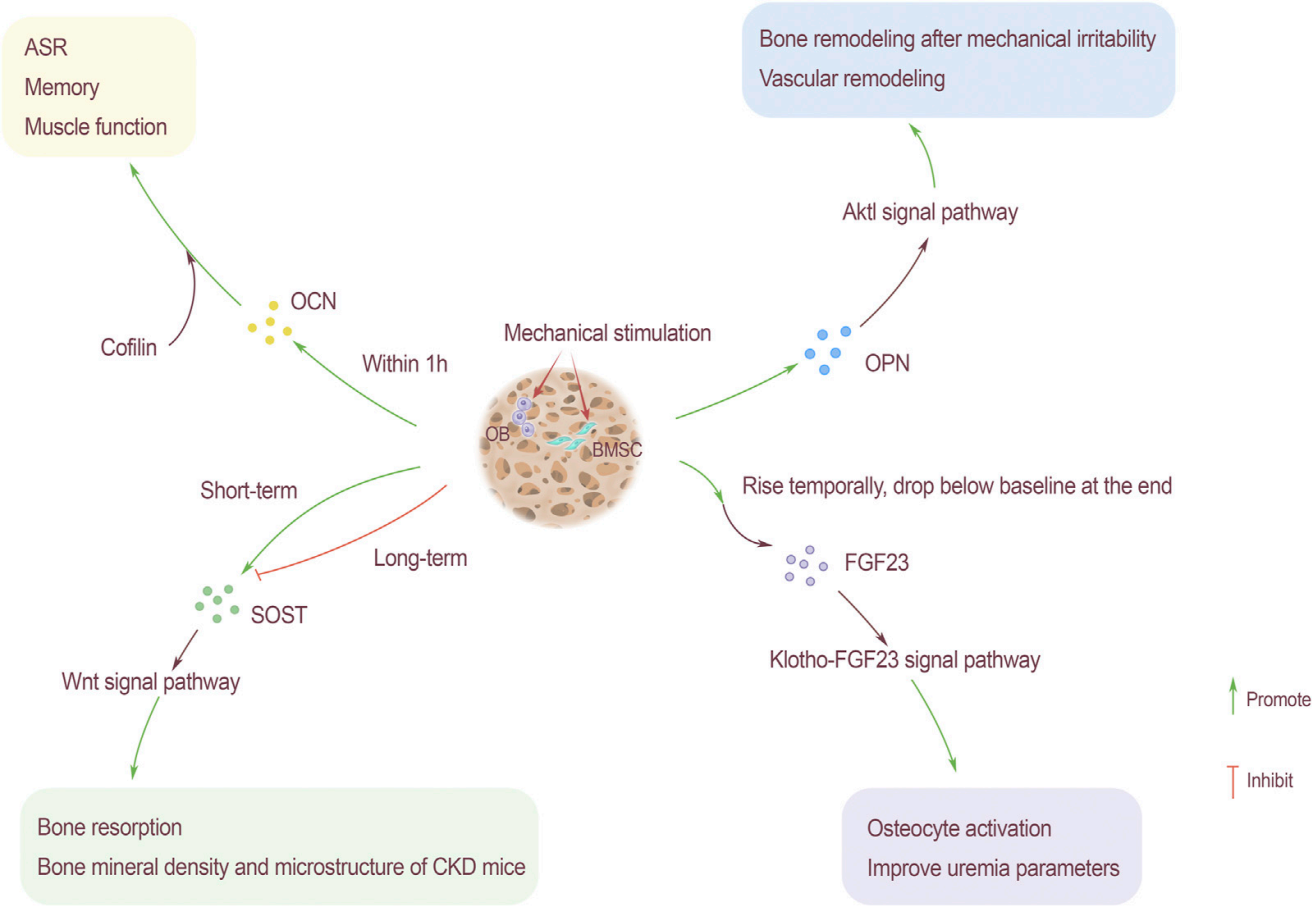
Mechanical stimulation as an influencing factor of bone-derived factors. OCN rises within 1 hour after mechanical stimulation, and through the participation of actin binding protein cofilin, it promotes ASR, memory and muscle function. Mechanical stimulation also promotes the content of OPN, the Akt1 signaling pathway can promote bone remodeling caused by mechanical stress and hypertension-related vascular remodeling. The relationship between SOST content and mechanical stimulation is that SOST rises sharply after short-term mechanical stimulation and then drops below the baseline level. Long-term stimulation slows down the increase of SOST, which has an inhibitory effect. SOST promotes bone resorption and improves the bone density and microstructure of CKD rats through the Wnt signaling pathway. FGF23 rises briefly after receiving mechanical stimulation, and then drops to the baseline level slowly. Through Klotho-FGF23 signaling pathway, the activation of bone cells is enhanced and parameters of uremia will be improved.

The circulating level of OCN will increase with exercise. The level of OCN increases in high-intensity intermittent exercise (Hiam et al., 2021). Changing regulation of glucose to mobilize bones, finally complete exercises. This change has nothing to do with gender. For adolescents, people who regularly participates in physical exercise show higher levels of OCN(Chahla et al., 2015). The increase of OCN level during exercise can promote acute stress response (ASR) (Berger et al., 2019), improve memory and enhance muscle function. For bone spine animals, ASR is related to the survivability in hostile environments such as the wild (Figure 3).

The homeostasis of bone is very important to the body’s calcium and phosphorus balance. OPN is an important phosphorylated glycoprotein, the fluctuations of which play a key role in bone formation and bone resorption. To maintain bone homeostasis, moderate exercise and physical activity are vital factors. Knockout OPN gene can inhibit mechanical stress bone remodeling in mice. In obese people and adipose tissue of mouse, OPN is widely up-regulated. After exercise to lose weight, the level of OPN in serum appears to decrease. The reason may be the body vibration caused by exercise or physiological factors, rather than the loss of fat (Garfield et al., 2016) (Figure 3).

As one of the important bone-derived factors, the fluctuations of FGF23 are critical to the normal operation of the kidney, small intestine, cardiovascular and other organs. Over-distance walking will cause the serum FGF23 level to increase temporarily, but it will decrease by the end of the exercise, and return to baseline level soon after the game (Kerschan-Schindl et al., 2021). This transient high expression of FGF23 may be related to the short-term bone metabolism uncoupling signal, which is triggered by the endocrine system after excessive exercise. Osteocytes are the mechanical sensors of bone, the increase of FGF23 level may be related to the activation of bone cells induced by exercise. In addition, exercise also helps in the treatment of kidney-related diseases. Resistance training for patients with stage II chronic kidney disease can improve uremia parameters and the klothof-FGF23 signaling pathway, thereby alleviating the progression of the disease (Corrêa et al., 2021) (Figure 3).

SOST is mainly secreted by bone cells, which are the main cells responsible for mechanical signal transduction. Therefore, SOST is regulated by mechanical stress, and exercise has a regulating effect on SOST. Under the stimulation of short-term exercise, the blood will release the synthesized SOST, which may be related to the physiological regulation of the kidneys. By reducing excretion, it increases the reabsorption of SOST by the renal tubules (Pickering et al., 2017). After endurance training, the concentration of serum SOST will increase, and the changes in SOST caused by exercise can be used as the basis for exercise metabolism monitoring (Jürimäe et al., 2021). The increase in serum SOST after exercise may be due to the release of SOST anchored in bone cells from bone cells into the blood, rather than the increase in SOST gene expression in a short period of time, and the increase in blood flow to bone caused by exercise (Kouvelioti et al., 2018). Exercise training slowed down the increase in serum SOST, the bone formation rate did not change but bone resorption was improved after intervention. Exercise can improve the density and microstructure of bone in CKD rats by inhibiting SOST, but it will not change the serum mineral content (Liao et al., 2019). After completing general short-term acute exercise, serum SOST increases acutely, and then decreases to normal levels or even lower. Long-term exercise makes the bones adapt to mechanical stress, the response of bone cells to exercise and serum SOST are both reduced. Long-term reduction of mechanical stress increases serum SOST, which is consistent with the regulation of bone metabolism by Wnt signaling pathway (Figure 3).

## Conclusion

The existing research shows that the actuating scope of bone-derived secretory factors is not limited to bones. In fact, it displays more hormonal properties and can regulate organ activitivies through special receptors. It plays a role in multiple systems and is closely related to the body’s metabolic process. According to different secreting cells, bone-derived factors can be divided into two categories, secreted by specific cells or multiple cells. Different bone-derived factors have differences in structures and locations, and these differences will affect their effects. Multiple bone-derived factors may simultaneously regulate the same organ or system. Organs and systems will also give feedbacks to the factors after receiving the signals, which further reflect the characteristics of their hormones. The effect of mechanical stimulation on bone will affect the secretion of bone-derived factors, and this effect will be reflected in the regulation of organs. Although it is not yet known that whether we could change the amount of bone-derived protein secretion by exerting mechanical stimulation to intervene in the treatment for dieases. However, the cross-organ regulation of bone-derived protein can provide theoretical bases for them.
